# Photonic jet lens

**DOI:** 10.1038/s41598-019-41193-2

**Published:** 2019-03-18

**Authors:** Sylvain Lecler, Stephane Perrin, Audrey Leong-Hoi, Paul Montgomery

**Affiliations:** ICube, University of Strasbourg - CNRS (UMR 7357), 300 Bd Sébastien Brant, 67412 Illkirch, France

## Abstract

Microsphere-assisted microscopy currently benefits from a considerable interest in the microscope-research community. Indeed, this new imaging technique enables the lateral resolution of optical microscopes to reach around *λ*/5 through a full-field and a far-field acquisition while being label-free. Despite the photonic jet clearly not being a relevant concept to justify the super-resolution phenomenon, we show here how it can be used to predict imaging formation and performance such as the image position and the microsphere magnification. This study allows a better understanding of the experimental measurements that have been observed over the last decade and that will be observed in coming years, through numerical simulations using different optical and geometrical parameters.

## Introduction

**Figure 1:**
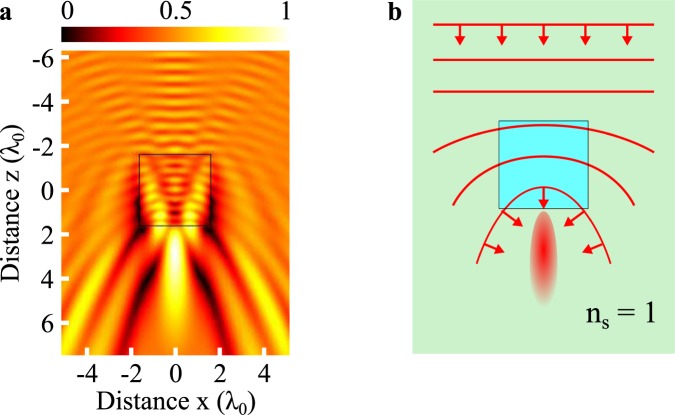
Generation of a photonic jet through a square particle. (**a**) The normalised modulus of an electric field interacting with the particle was retrieved using a finite element method in the TE mode. The size and the refractive index of the particle are 3 *λ*_0_ and 1.3 respectively, with *λ*_0_ being the wavelength in air. **(b)** A scheme of wavefront distortion (red solid line) of the unitary plane wave is represented considering the continuity of the tangential component of the electric field in the particle and in the surrounding air medium, resulting in a photonic jet. Red arrows represent the wavefront vectors.

Supposedly known since 1908, the rigorous and complete Lorenz-Mie theory predicts the interaction of light with spherical particles^1^ and visible light scattered by dielectric microspheres^2^ in the far field. However, over the last decades, the enhancement of computing power has made it possible to discover a focusing phenomenon in the near field of dielectric particles. Indeed, light is concentrated in a sub-diffraction-limit progressive beam known as photonic jet (PJ)^3,4^. This phenomenon is not specific to dielectric spherical-shape particles. As a matter of fact, a square glass particle interacting with a plane incident wave is also able to generate a PJ (Fig. 1a)^5^. Moreover, at this scale, propagation of light does not obey the classical laws of geometrical optics. The focusing effect is not only due to refraction of light by the particle but also to diffraction. The propagation of light inside the particle as well as around the particle must thus be considered in order to predict the generation of the PJ. Delayed inside the particle, the electromagnetic incident wave is also delayed continuously around the particle due to the continuity of the tangential electric field components, leading to a wavefront curvature as illustrated in Fig. 1b. In this work, only circular or cylindrical particles are considered.

**Figure 2:**
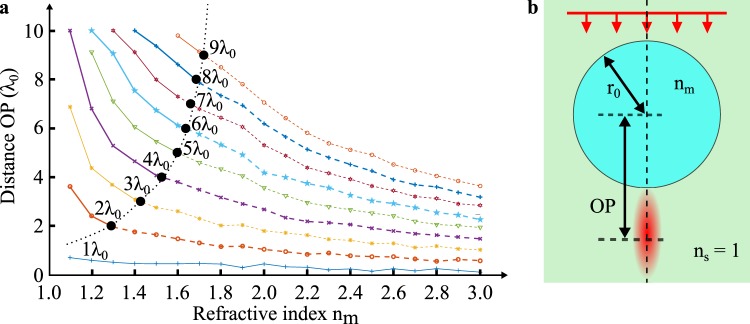
Axial position of the photonic jet irradiance peak. (**a**) The position of the photonic jet spot *P* is estimated as a function of the microsphere refractive index *n*_*m*_ for different radii *r*_*o*_. **(b)** In the Lorenz-Mie model, the microsphere is illuminated by an incident plane wave with a wavelength of *λ*_0_ in air (*n*_*s*_ = 1.0) in order to retrieve the focusing phenomenon. Nine radius-dependent curves are represented, going from *r*_*o*_ = 1 *λ*_0_ to *r*_*o*_ = 9 *λ*_0_, with an increment of 1 *λ*_0_. The distance *OP* and *r*_*o*_ are expressed in wavelength *λ*_0_. The black dots show the position of the point *P* on the surface of the microsphere. For example, when *n*_*m*_ = 1.52 and *r*_0_ = 4.0 *λ*_0_, then *OP* = *r*_*o*_. Below, the PJ irradiance peak position is within the microsphere, *i.e*. $\overline{OP}\mathrm{\  < \ }{r}_{0}$.

The position and the width of the PJ irradiance peak depends on geometrical (*e.g*. the particle size) and optical (*e.g*. the particle material and the wavelength of light) parameters. Since the ray-tracing is not relevant to study PJ performance, a rigorous electromagnetic model based on the Lorenz-Mie theory must be implemented. Figure 2 represents the evolution of PJ axial position *P* from the centre *O* of a microsphere as a function of its refractive index *n*_*m*_ and for different radius *r*_*o*_. The wavelength of the plane wave incident on the microsphere is *λ*_0_ in air (*n*_*s*_ = 1.00). The distance *OP* and the microsphere radius *r*_*o*_ are expressed in wavelength *λ*_0_ due to the invariant Maxwell equations by spatial scale modification. At a given radius *r*_*o*_, the higher the refractive index *n*_*m*_, the smaller the distance *OP*, meaning the closer the PJ is to the center *O*. Nevertheless, at a given microsphere refractive index *n*_*m*_, an increase in the radius *r*_*o*_ moves away the PJ. The black points indicate that the focus point *P* is on the microsphere interface, *i.e. OP* = *r*_*o*_. Through its asymptotic tendency (represented by the black dashed curve), the PJ appears always inside the microsphere regardless of the radius *r*_*o*_ when the refractive index is higher than 1.8^3^. It can be noted that using Snell’s law, the refractive index is higher and equals 2.0 for larger microsphere sizes^6^.

In 2011, Wang *et al*. showed that dielectric microspheres do not only display the property of concentrating light in the near field, but can also perform a full-field super-resolved image in the far field^7^. In other words, the microsphere is not merely a lens-like component focusing a near-field beam but it is also able to form an image. Amongst the numerous methods allowing microscopy to become nanoscopy^8^, microsphere-assisted microscopy is a new imaging technique which provides the advantage of being full field, label free, and easy to implement. Miniature glass spheres have thus, once again, become “super-lenses” in microscopy, 350 years after van Leeuwenhoek used a single sphere of around 1 mm in size to visualize the first bacteria^9^. Although the phenomenon is currently still not explained, many papers studied performance of this imaging technique in 2D^10–13^ and in 3D^14–16^. According to the Mie theory prediction, the PJ generation by a microsphere is not relevant to justify this resolution improvement in the imaging process. Although the size of the focus spot overcome the diffraction limit, the full width at half maximum (FWHM) of the near-field beam waist is around a third of the wavelength^17^ which is higher than the lateral resolution obtained experimentally, *i.e*. around *λ*_0_/7^10^ (or even *λ*_0_/17^18^ using overestimated resolution criteria based on the gap measurement of features^19^). However, the PJ phenomenon can explain the imaging formation in microsphere-assisted microscopy, *i.e*. the nature of the image, the position of the image plane and the lateral magnification provided by the microsphere. Indeed, according to many studies on the PJ prediction^3,20^, the imaging process in microsphere-aided microscopy can be easily addressed by considering the sphere as a *photonic jet lens*.

**Figure 3:**
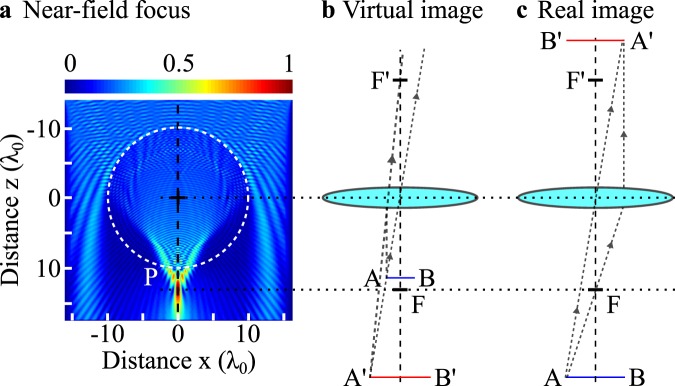
Concept of photonic jet lens. (**a**) A glass microsphere forms a photonic jet where its irradiance peak *P* can be seen as the object focal point *F* of the microsphere. In geometrical optics, this assumption leads to the formation of (**b**) a virtual image when the object is placed between *P* and the microsphere, and (**c**) a real image when the object is placed further than *P*. The 2D FEM simulation was performed using an illumination wavelength *λ*_0_ of 500 nm in air. The refractive index of the 10 *λ*_0_-radius glass microsphere is 1.52, giving *OP* = 12.5 *λ*_0_.

In this manuscript, the prediction of the image formation, *i.e*. the image nature, the image position and the lateral magnification, is descripted in microsphere-assisted microscopy by assimilating the microsphere as a classical lens with geometrical optics. Indeed, due to the plane incident wave which can be assimilated to an infinitely distant object, the irradiance peak *P* of the PJ generated by the microsphere can be seen as the object focal point *F* of a classical lens (Fig. 3a). The PJ generated by a soda lime glass microsphere has been numerically reconstructed using a plane incident wave in the TE mode. Assuming $\overline{OP}$ = $\overline{OF}$ and by means of Fig. 2, the nature of the image $\overline{A^{\prime} B^{\prime} }$ of an object $\overline{AB}$ can then be retrieved (*e.g*., virtual imaging as schematically shown in Fig. 3b or real imaging as shown in Fig. 3c). Furthermore, a qualitative evolution of both the image position and magnification factor from the microsphere can be predicted as a function of the position of the object plane as well as the size and the refractive index of the microsphere.

## Results

**Figure 4:**
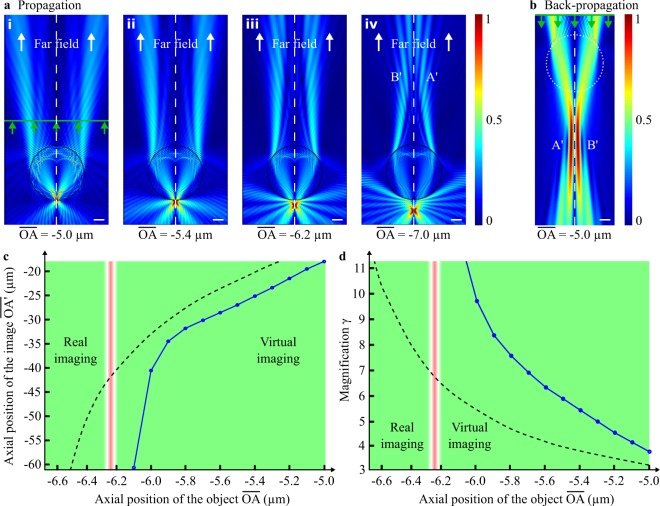
Influence of the object plane position on the image formation. The electric field from two out-of-phase point sources *A* and *B*, spaced 400 nm apart in air, is first propagated through the microsphere where **(a)** the normalised modulus of the electric field is represented for different axial positions $\overline{OA}$. When, **(a.i,a.ii)** the object $\overline{AB}$ is above the PJ spot, **(b)** the back-propagation operation in the surrounding medium (from green line) is required to retrieve the potential virtual images *A*′ and *B*′. When, **(a.iii)** $\overline{AB}$ is in the intermediate axial range around the PJ, the image is formed at infinity. Finally, when **(a.iv)** the object $\overline{AB}$ is clearly below the PJ irradiance peak, the image becomes real. White-dotted lines represent the optical axis of the simulation and green arrows represent the direction of the time-reversed propagation. White scale bars represent 2 μm. **(c)** The image position and **(d)** the magnification are calculated as a function of $\overline{OA}$. The simulations have been performed with a wavelength *λ*_0_ of 500 nm using a 1.52-refractive-index microsphere with a radius of 10 *λ*_0_. The transverse distance $\overline{AB}$ is 400 nm. Black-dotted lines represent the evolution curves of the image plane position and the magnification using geometrical optics relations with a 5 μm-radius glass ball lens^22^. This assumption shows a similar behaviour of the curves although the values are different at this scale (*e.g*. the focal length of the ball lens equals 7.30 μm and *γ* = n/(2 − n) = 3.17 when $\overline{OA}$ = −5.0 μm).

The influence of the object plane position on the image formation was firstly analysed. Therefore, the object has been displaced along the optical axis, and both the image plane position $\overline{OA^{\prime} }$ and magnification factor *γ* were estimated. Furthermore, the comparison with the classical geometrical laws was studied. The results are shown in Fig. 4. The simulation was implemented in air (*n*_*s*_ = 1.00) using here two emitting-point sources *A* and *B* as mentioned in Methods section (unlike the previous simulations where a plane wave illuminated the microsphere for finding the focusing spot). The object $\overline{AB}$ is placed at different axial position $\overline{OA}$ under a soda-lime-glass microsphere (*n*_*m*_ = 1.52) having a radius *r*_*o*_ of 5 μm. The wavelength *λ*_0_ of the emitting electric field being here of 500 nm, *r*_0_ equals thus 10 *λ*_0_ and the PJ spot is outside the microsphere at $\overline{OP}$ = −6.25 μm = −12.5 *λ*_0_ (Fig. 2a). When the point sources are placed between the last dioptre of the microsphere and the PJ spot, *i.e*. $\overline{OA}$ = [−5.0 μm; −6.2 μm] (Fig. 4a.i and a.ii), the image is formed above the microsphere and is thus virtual (Fig. 4b). In this case, the further the object $\overline{AB}$ is from the microsphere, the further away is the position of the image $\overline{A^{\prime} B^{\prime} }$ (Fig. 4c). Moreover, the magnification remains positive and increases steadily as shown in Fig. 4d. An asymptotic behaviour of the two curves appears when the object plane meets the focusing plane, *i.e*. when $\overline{OA}$ = $\overline{OP}$. At this particular position, the image is located at infinity in the far field (Fig. 4a.iii). This phenomenon can also be found over a short distance ($\overline{OA}$ = [−6.2 μm;−6.3 μm]) which can be assimilated as the range of the Gouy phase shift^21^. In this range, the magnification *γ* goes on to increase continuously until reaching infinity. This asymptotic tendency to infinity is similar in geometrical optics^22^ (black-dashed curve) when $\overline{OA}$ = $\overline{OF^{\prime} }$:

1 $${\gamma }_{geo}=\frac{1}{\frac{\overline{OA}}{\overline{OF^{\prime} }}-1}$$

Finally, beyond the *P* point position, the image $\overline{A^{\prime} B^{\prime} }$ appears real and is formed above the microsphere as shown in Fig. 4a.iv. The distance $\overline{OA^{\prime} }$ is then positive and *γ* becomes negative.

**Figure 5:**
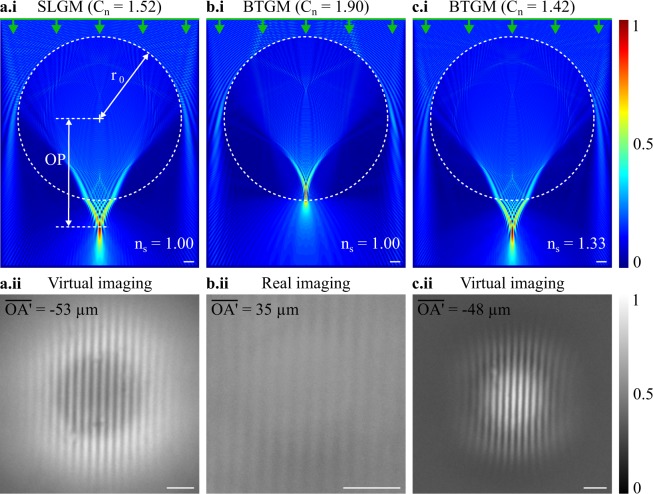
Image formation depending on the refractive index contrast. *C*_*n*_ between the microsphere and the surrounding medium. A soda lime glass microsphere SLGM (*n*_*m*_ = 1.52), **(a.i)** generating a photonic jet outside the microsphere ($\overline{OP}\mathrm{\  > \ }{r}_{o}$) in air (*n*_*s*_ = 1.00), thus **(a.ii)** a virtual image occurs. Whereas, using a barium-titanate-glass microsphere BTGM (*n*_*m*_ = 1.90) in air, **(b.i)** the focus spot *P* appears inside the microsphere, **(b.ii)** providing a real image. Replacing the air immersion medium by water (*n*_*s*_ = 1.33) allows **(c.i)** the photonic jet position to be further than the microsphere interface ($\overline{OP}\mathrm{\  > \ }{r}_{o}$) and **(c.ii)** the image to be once again virtual. In the three cases, the microspheres have a radius *r*_*o*_ of 16 μm. The photonic jet beams are simulated using a plane incident illumination with *λ*_0_ = 600 nm. The super-resolved images were experimentally measured using a 600-nm-period Ronchi grating placed against the microspheres ($\overline{OA}$ = *r*_*o*_). White scale bars represent 2 μm.

A modification of the microsphere refractive index *n*_*m*_ provides also an influence on the imaging formation. Indeed, an increase in *n*_*m*_ results in the focusing point *P* being closer to the sphere interface. Furthermore, for *n*_*m*_ being higher than 1.8, we have shown that the focusing spot *P* is inside the sphere regardless to the microsphere size (Fig. 2a). In this situation, $\overline{A^{\prime} B^{\prime} }$ should thus always be real. We validated this assumption using two microspheres having different refractive index, *i.e*. a soda-lime-glass microsphere (*n*_*m*_ = 1.52) and a barium-titanate-glass microsphere (*n*_*m*_ = 1.90), and a similar diameter (*r*_*o*_ = 16 μm). A 600-nm-period positive Ronchi grating (Aluminium features on glass wafer) was used as object and placed against the microsphere surface ($\overline{OA}$ = *r*_*o*_). When the PJ spot *P* is outside the microsphere (simulation in Fig. 5a.i), the latter generates a virtual image (experiment in Fig. 5a.ii). Whereas, when *P* is inside the microsphere (simulation in Fig. 5b.i), the image appears to be real and less contrasted (experiment Fig. 5b.ii). It can be mentioned that, in geometrical optics, the effective focal length of a ball lens is smaller than the radius only when the refractive index is superior to 2.0^22^. This image process has already been experimentally presented in microsphere-assisted microscopy using both a 30 *λ*_0_-radius barium-titanate-glass microsphere and a 35 *λ*_0_-radius polystyrene microsphere (*n*_*m*_ = 1.59)^12^. Maslov and Astratov have also demonstrated the influence of the refractive index on the image process through simulations^23^. It has been shown that a microsphere having a radius of 3.2 *λ*_0_ and a refractive index of 1.4, performs virtual imaging when the object is at 3.3 *λ*_0_ from the microsphere interface, *i.e*. above the focusing spot *P* position. And, a 1.9-refractive-index microsphere results in real imaging. These results confirm our assumption which considers the microsphere as a *photonic jet lens*.

Placing now the 16 μm-radius barium-titanate-glass microsphere in water (*n*_*s*_∼1.33) provides a PJ which is below the microsphere (simulation in Fig. 5c.i) and a virtual image occurs (experiment in Fig. 5c.ii). As a matter of fact, according to Mie theory, a sphere with a refractive index *n*_*m*_, embedded in a surrounding medium of refractive index *n*_*s*_ and illuminated by a beam having a wavelength *λ*_0_ in air, is equivalent to a sphere having a refractive index of *n*_*m*_/*n*_*s*_ in air and being illuminated by an electric field wavelength of *λ*_0_/*n*_*s*_^2^. We can hence deduce that a barium titanate microsphere immersed in water (*n*_*s*_∼1.33) is equivalent to a silica glass microsphere (*C*_*n*_ = *n*_*m*_/*n*_*s*_∼1.90/1.33∼1.42) having an equivalent size parameter but illuminated with a wavelength *λ*_0_ divided by *n*_*s*_. Numerical simulations and experiments in Fig. 5a,c confirm this concept. The clear interest of immersion microsphere-assisted microscopy is thus to benefit from a smaller wavelength as in classical microscopy. It could be mentioned that considering the influence of the refractive index of the surrounding medium, new simulations are hence not required.

**Figure 6:**
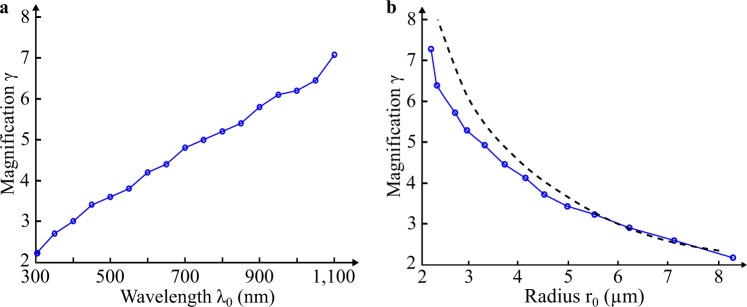
Magnification factor *γ* of the microsphere depending on optical and geometrical parameters. (**a**) *γ* is expressed as a function of the emitting wavelength *λ*_0_. The object $\overline{AB}$ was spaced 400 nm apart and placed against the microsphere having a refractive index of 1.52 ($\overline{OA}$ = *r*_0_ = 5 μm). For example, *γ* = 3.6 when *λ*_0_ = 500 nm. **(b)** *γ* is then calculated as a function of the microsphere radius *r*_0_ using Eq. 2 with *λ*_0_ = 500 nm. Black-dashed line represents a curve fitting of *γ* using geometrical optics defined as *γ* = *κ*/*f*′ where *f*′ is the focal length of a ball lens and *κ* a constant value equalling 25.6 μm in this case^22^.

The influence of the probing wavelength on the magnification *γ* provided by the microsphere is also analysed. Here, the object $\overline{AB}$ is placed in contact with a 10 *λ*_0_-radius microsphere. The simulations are performed for wavelengths *λ*_0_ in air within the visible range. As shown in Fig. 6a, the longer the wavelength *λ*_0_, the higher the magnification *γ* following a linear evolution. Through the PJ evolution according to the wavelength (Fig. 2), the increase of *γ* can be understood. Indeed, for long wavelengths, the PJ appears closer to the microsphere and thus to the object $\overline{AB}$, consequently making the magnification higher. In accordance, the image distance increases for high wavelengths. It should be noted that, here, the wavelength dependence of the microsphere refractive index^24^ is not considered, *i.e. n*_*m*_ = 1.52 regardless the wavelength in the simulations. Thus, the generation of chromatic aberrations does not come from the material dispersion, *i.e. n*_*m*_(*λ*_*i*_), but from both the shape and the size of the microsphere, as retrieved in optical fibre telecommunications.

Measuring the magnification *γ* as a function of the radius *r*_*o*_ of the microsphere could provide wrong interpretations of the results. Indeed, changing *r*_*o*_ means that the distance $\overline{OA}$ is also modified. However, due to the scale invariance from the Maxwell equations in the spatial domain, changing the sphere radius *r*_1_ for a constant source wavelength in air *λ*_1_ is equivalent to changing the source wavelength in air *λ*_2_ with a constant sphere radius *r*_2_ with:

2 $$\frac{{r}_{1}}{{\lambda }_{1}}=\frac{{r}_{2}}{{\lambda }_{2}}$$

An increase in the wavelength is interpreted as a decrease in the radius of the microsphere. Thus, the magnification *γ* of the microsphere has been expressed as a function of its radius *r*_0_ in Fig. 6b from the previous results (Fig. 6a). As in geometrical optics (black-dashed curve)^22^, the magnification *γ* clearly decreases when *r*_0_ increases. Indeed, a sphere radius increase leads to a longer distance between the PJ and the microsphere (as well as the object $\overline{AB}$ located at their default position in a plane tangent to the sphere) and hence, a reduction of *γ*. Reciprocally, when *r*_0_ decreases, the focus point *P* becomes closer to the object plane (Fig. 2), giving an improvement of *γ* until it reaches an infinite value and an image located at infinity in the far field. By this means, the magnification should be considered to enable the imaging of the virtual image by the microscope objective. However, there is no interest in large magnification. Indeed, a too large magnification requires spheres with a small diameter, reducing the field of view and taking away the virtual image from the sphere.

## Discussion

To date, the super-resolution phenomenon through microsphere-assisted microscopy has not been explained and is still in discussion. In this manuscript, it has been shown that the photonic jet does not form the basics of the lateral resolution enhancement. Indeed, the photonic jet effect (with a FWHM of around *λ*_0_/3 in air) is not relevant to justify the observed resolution. The evanescent waves appear yet to be more pertinent to justify the lateral resolution enhancement and this approach is supported by several recent papers^25–27^. Moreover, the role of the coherence should also be considered^10^. If the role of the evanescent waves is confirmed, we must underline that the photonic jet is a phenomenon based on propagative waves and can describe the imaging process. Nevertheless, it cannot be excluded that the imaging process could be slightly different through the evanescent wave approach, providing a shift in the focus point. Such a hypothesis could explain the observation of two image planes^13,23^. One of them would be the image of the propagative waves, *i.e*. small k wave vector, and the second one, the image of the evanescent waves, *i.e*. large k wave vector. However, this is still only a hypothesis. The recent results concerning phase measurements and their exploitation for 3D reconstruction will be an opportunity for further investigation^16^.

## Conclusion

This paper shows that photonic jet physics has to be considered in order to understand the imaging formation in microsphere-assisted microscopy. At this scale, the microsphere cannot be seen as a classical lens due to its small radius which makes the focus generation not only related to the refraction of light but also to the diffraction of light. However, considering the photonic jet irradiance peak as the focus point, we can deduce the nature of the image, *i.e*. real or virtual, as well as the tendency of the image position and the magnification from the microsphere. In this manuscript, simulations and experiments of performance of microsphere-assisted microscopy illustrate this phenomenon and allows the analogy with geometrical optics to be made. Indeed, it has been shown that placing the object plane before or after the photonic jet spot leads to a virtual or a real image, respectively. Furthermore, the magnification factor provides a behavior which is similar to geometrical optics. This similitude between geometrical optics and the *photonic jet lens* laws has been further highlighted through the study of the near-field focusing spot position by changing the refractive index of the microsphere and the surrounding medium. The use of high-refractive-index microspheres and immersion medium is also discussed, giving an obvious enhancement of performance due to the smaller equivalent wavelength in the surrounding medium. This effect can also be retrieved in immersion optical microscopy. Finally, the microsphere imaging process has been analysed as a function of the illumination wavelength and the size of the microsphere.

## Method

**Figure 7:**
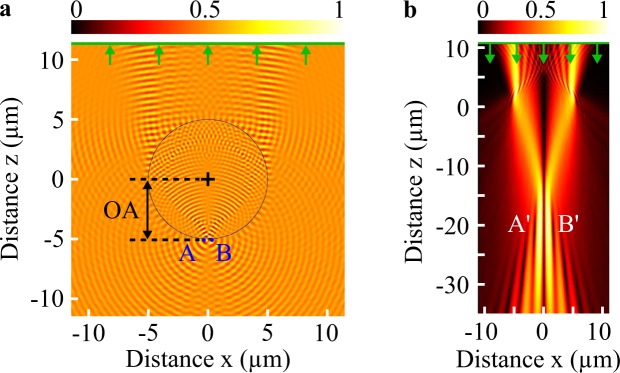
Method of the numerical analysis of microsphere-assistedmicroscopy. To retrieve the image position, the complex electric field from the out-of-phase emitting points *A* and *B* is first propagated through themicrosphere and maintained (green line on the top) to be then back-propagated in the surroundingmedium without the microsphere. (**a**) The real part of the electric field and (**b**) the normalized absolute value of the time-reversed electric field are represented. Here, *A* and *B* are placed between the microsphere and the irradiance peak of the photonic jet, leading to virtual images *A*0 and *B*0. The microsphere has a radius *ro* of 5 μm and a refractive index *nm* of 1.52. The wavelength *λ*_0_ is 500 nmand the surroundingmedium is air (*ns* = 1.0).

To demonstrate the relevance of the *photonic jet lens* concept, the performance of microsphere-assisted microscopy has been numerically computed using two rigorous numerical simulations in the TE mode. The propagation equations were solved using a 2D finite-element method (Comsol Multiphysics) with perfectly matched layers as radiation numerical boundary conditions. In this 2D model, the microsphere is reduced to a cylinder where the axis is along the y axis and is placed in a medium with a refractive index *n*_*s*_. The radius and the refractive index of the cylinder are *r*_*o*_ and *n*_*m*_, respectively. Two point sources *A* and *B*, serving as object, are placed symmetrically in the horizontal plane below the microsphere at an axial distance $\overline{OA}$. The dipoles *A* and *B* have a coherent phasor with a relative phase difference of pi in order to contribute to separate their images^10,15^. In the first simulation, the complex electric field from *A* and *B* is transmitted through the microsphere and the far field is recorded (Fig. 7a). Second, if the image is virtual, this complex electromagnetic field is back-propagated (or time-reversal propagated) in the surrounding medium *n*_*s*_ without the microsphere (Fig. 7b). It should be noted that, if the image is real, only the propagation operation is needed. The images *A*′ and *B*′ of the two point sources *A* and *B* are recovered by finding the maxima values of the modulus of the electric field which is proportional to the irradiance of light. Finally, the axial position $\overline{OA^{\prime} }$ of the image and the magnification *γ* (defined as the ratio between transversal distances $\overline{A^{\prime} B^{\prime} }$ and $\overline{AB}$) are determined. In the experiments, an objective lens above the microsphere collects the far field from the object and relays it onto a camera sensor. Obviously, the performance of the objective lens must be significant to resolve the two point sources *A*′ and *B*′.

Moreover, experiments has been performed by introducing soda-lime-glass or barium-titanate-glass microspheres in a classical optical microscope, *i.e*. below the objective lens of the microscope (×20, NA = 0.4, Zeiss) and onto the object surface. The microspheres were illuminated by a light source having a central wavelength of 600 nm. The object consists of a Ronchi grating having a period of 600 nm with a groove of 300 nm and feature height of 100 nm. The images (real or virtual depending on both the sphere material and immersion conditions) were collected by matching the microscope object plane with the microsphere image plane. Finally, the images were recorded by a camera without implementing image processing algorithms. It should be mentioned that the microscope alone is able to resolve two details having a separation distance of at least 800 nm.
